# Goodpasture’s syndrome with absence of circulating anti-glomerular basement membrane antibodies: a case report

**DOI:** 10.1186/s13256-016-0984-6

**Published:** 2016-07-27

**Authors:** Rui Fernandes, Sara Freitas, Pedro Cunha, Gloria Alves, Jorge Cotter

**Affiliations:** 1 Internal Medicine Department Guimarães, Centro Hospitalar do Alto Ave Rua dos Cutileiros Creixomil, 4810 Guimarães, Portugal; 2 Life and Health Science Research Institute (ICVS) School of Health Science, University of Minho, Braga, Portugal

**Keywords:** Anti-GBM antibodies, GBM, Glomerular basement membrane, Goodpasture’s syndrome, Glomerulonephritis, Renal failure, Rapidly progressive glomerulonephritis, Case report

## Abstract

**Background:**

Goodpasture’s syndrome, a rare disease, is an organ-specific autoimmune disease mediated by anti-glomerular basement membrane antibodies. Its pathology is characterized by crescentic glomerulonephritis with linear immunofluorescent staining for immunoglobulin G on the glomerular basement membrane. Although rare, a few cases with absence of circulating anti-glomerular membrane antibodies have been described.

**Case presentation:**

The objective of this clinical case report is to describe and discuss a case of a 27-year-old white man who was hospitalized with a 1-year history of weight loss and a 1-month history of hemoptysis, with aggravation  the day before, having developed dyspnea and cough in the previous 24 hours. An analytical study showed normocytic normochromic anemia with a hemoglobin level of 7.2 g/dL and leukocytosis with normal renal function and coagulation times. A blood transfusion was performed without complications. Chest computed tomography revealed a reticulonodular infiltrate of both lungs. Bronchoscopy showed no apparent lesions. Sputum cultures, rapid urine antigens for *Legionella pneumophila* and *Streptococcus pneumoniae*, studies for *Influenza*, virologic markers and serologic studies for autoimmunity were all negative. At the end of the tenth day his general state deteriorated with fatigue, hematuria, and in 3 days he developed aggravation of renal function with recurrent hemoptysis and anemia. Immunosuppression with daily prednisolone 1 g administered intravenously was initiated. An urgent bronchoscopy showed no lesions. A kidney biopsy showed fibrinoid necrosis and cellular crescents. Immunofluorescence revealed a linear immunoglobulin G deposition compatible with Goodpasture’s syndrome. Immunosuppressive therapy with daily cyclophosphamide 120 mg orally was added. Subsequently he was transferred to a referral center at which 21 sessions of plasmapheresis and four sessions of hemodialysis were performed with good response; he currently has no need of hemodialysis.

**Conclusions:**

The absence of circulating anti-glomerular basement membrane antibodies in Goodpasture’s syndrome adds complexity to the diagnosis creating an unusual setting in a rare disease. In our case a kidney biopsy was essential for diagnosis and clinical approach. Studies have shown that early aggressive therapy leads to an improved prognosis. Physicians should consider tissue diagnoses such as bronchoscopy and kidney biopsy in pulmonary renal syndrome.

## Background

Goodpasture’s syndrome (GS) is a rare disease, identified by Ernest Goodpasture [1]. It is an organ-specific autoimmune disease that is mediated by anti-glomerular basement membrane (anti-GBM) antibodies and its pathology is characterized by crescentic glomerulonephritis with linear immunofluorescent staining for immunoglobulin G (IgG) on the glomerular basement membrane (GBM). Its typical presentation is acute renal failure due to rapidly progressive glomerulonephritis, as well as pulmonary hemorrhage, which may be life-threatening [2, 3]. It is estimated that the incidence of GS is one case per million per year; however, it is responsible for acute renal failure in approximately 20 % of all cases of rapidly progressive or crescentic glomerulonephritis [4]. In the 1950s, Krakower and Greenspon [5] identified the GBM as the antigen. The titer of circulating autoantibodies is considered a measure of disease severity correlating with renal outcomes [3].

The objective of this clinical case report is to describe and discuss the case of a young man who presented with acute pulmonary and renal involvement with atypical features of GS.

## Case presentation

A 27-year-old white man was hospitalized on 17 September 2014 with dyspnea and cough, which had started the day before, and a 1-month history of small-volume hemoptysis, which had aggravated in the last 24 hours. He also reported a history of weight loss, corresponding to 12 % of his corporal mass in the past year, but no other accompanying symptoms. He reported a cigarette smoking habit of 4 pack years, having occasionally smoked recreational drugs (cannabis). He was also professionally exposed to chemical toxics: thinners. There was no previous history of renal or pulmonary disease. No family disease was known and he had no other relevant findings in his past medical history.

**Fig. 1:**
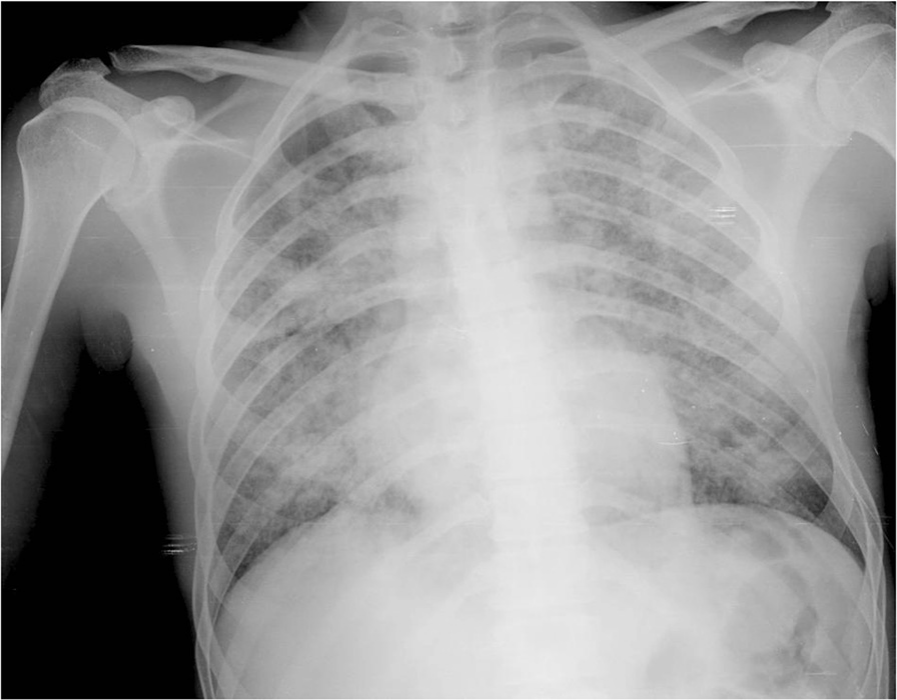
Chest X-ray performed at admission. The X-ray displayed bilateral pulmonary diffuse infiltrates

**Fig. 2:**
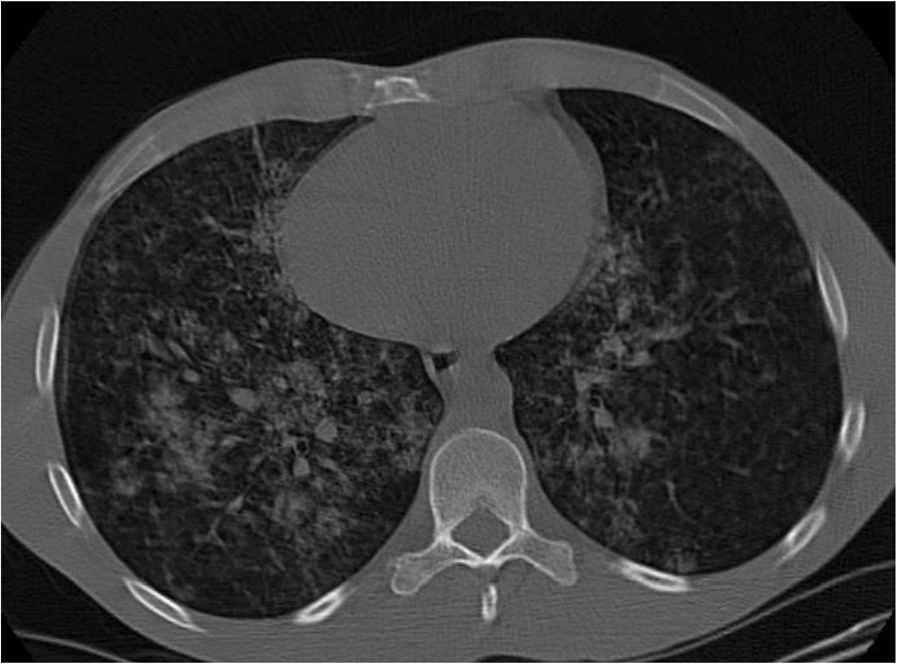
Chest computed tomography scan. The chest computed tomography scan showed bilateral multilobular diffuse infiltrates

On examination at the time of admission he was apyretic, his respiratory rate was 29 breaths/minute, pulse rate was 118 beats/minute, and blood pressure (BP) was 151/86 mmHg. We observed pallor of his skin. Pulmonary auscultation revealed rales in his right hemithorax and left base. A chest X-ray (CXR) showed bilateral infiltrates (Fig. 1). An analytical study (Table 1) showed a normocytic normochromic anemia with a hemoglobin level of 7.2 g/dL and leukocytosis with normal renal function and coagulation times. His erythrocyte sedimentation rate was 34 mm/hour and procalcitonin level was 0.309 ng/mL at the time of admission. A urine analysis had no erythrocyturia or eosinophiluria. His oxygen saturation was 96.8 % on 21 % oxygen with no signs of carbon dioxide (CO_2_) retention and no electrolytic or acid-base changes. Chest computed tomography (CT) revealed a reticulonodular infiltrate of both lungs with extensive ground glass appearance (Fig. 2).

**Table 1:** Analytical evolution for the period of hospitalization

	Admission (17 September 2014)	Post-transfusion (2 blood units; 18 September 2014)	Recurrence of hemoptysis (27 September 2014)	Post-transfusion (29 September 2014)	Reference values
Hemoglobin (g/dl)	7.2	8.9	6.1	9.1	12–16
Leucocytes (10^3^/μL)	13.1	24.1	25.7	24.1	4.8–10.8
Neutrophils (10^3^/μL)	9.9	21.9	23.6	22.3	1.8–7.7
Eosinophils (10^3^/μL)	0.1	0	0	0	0.0–0.49
Lymphocytes (10^3^/μL)	2.5	1.4	0.6	0.6	1.0–4.8
Platelets (10^3^/μL)	364	477	449	221	150–350
Urea (mg/dL)	40.0	43.0	189	29	15–56
Creatinine (mg/dL)	0.97	0.78	4.55	5.13	0.6–1.3
C-reactive protein (mg/dl)	12.7	42.0	12.4	5.5	<3.0
Prothrombin time (seconds)	12.7	11.0	10.9	10.7	9.4–12.5
Activated partial thromboplastin time (seconds)	28.6	22.2	24.2	26.3	25.1–35.6

**Fig. 3:**
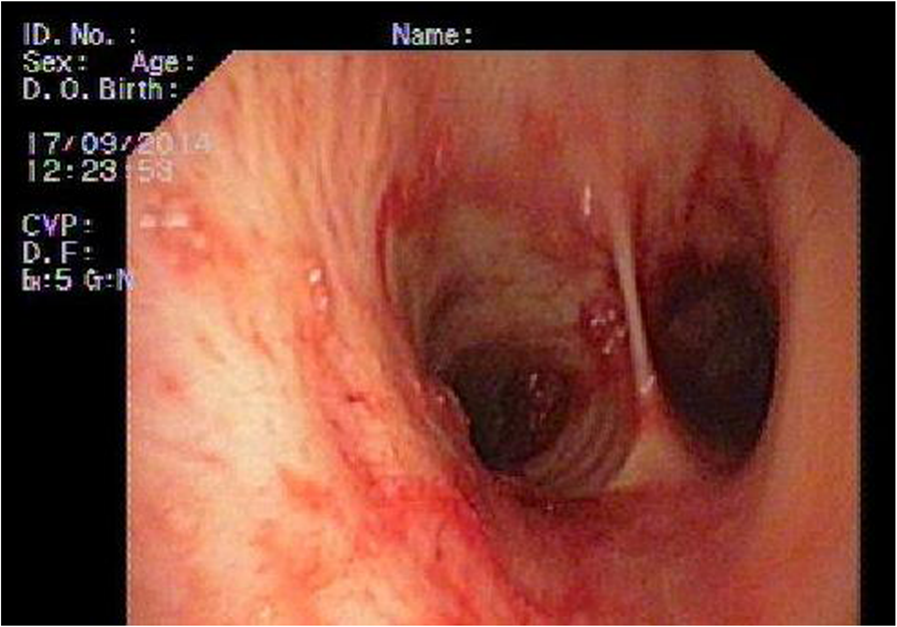
Bronchoscopy performed on the second day after admission. The examination revealed hematic traces throughout the tracheobronchial tree with no evident active bleeding site or clots

He was initially treated with aminocaproic acid 3 g orally every 8 hours and codeine 20 mg orally every 6 hours, having also completed 7 days of daily ceftriaxone 1 g administered intravenously and daily azithromycin 500 mg administered orally. Transfusion of two blood units was performed with an increase in hemoglobin count up to 8.9 g/dL (Table 1) without complications. His serum urea and creatinine levels were 43 mg/dL and 0.78 mg/dL, respectively, at this time. A bronchoscopy performed on the second day revealed hematic traces with no other alterations or apparent lesions (Fig. 3). On the fourth day of admission, he developed deterioration of ventilatory parameters and of respiratory rate (32 breaths/minute) needing oxygen supplement via high flow mask. Due to respiratory failure with a partial pressure of oxygen (pO_2_) of 48.3 mmHg and an oxygen saturation of 83.6 % on 100 % oxygen, he was transferred to our intensive care unit (ICU), but did not require mechanical ventilation. Sputum cultures were negative, as were the rapid urine antigen tests for *Legionella pneumophila* and *Streptococcus pneumoniae.* DNA analysis for *Mycobacterium tuberculosis* was negative, as were the studies for *Influenza A, Influenza B,* and *Influenza H1N1,* and the serologic markers for human immunodeficiency virus, hepatitis C and hepatitis B. Direct and indirect Coombs test were both negative. An echocardiogram was performed showing no significant findings. Serologic tests for auto-antibodies (antinuclear antibodies, anti-neutrophil cytoplasmic antibodies, and anti-GBM antibodies) were negative.

**Fig. 4:**
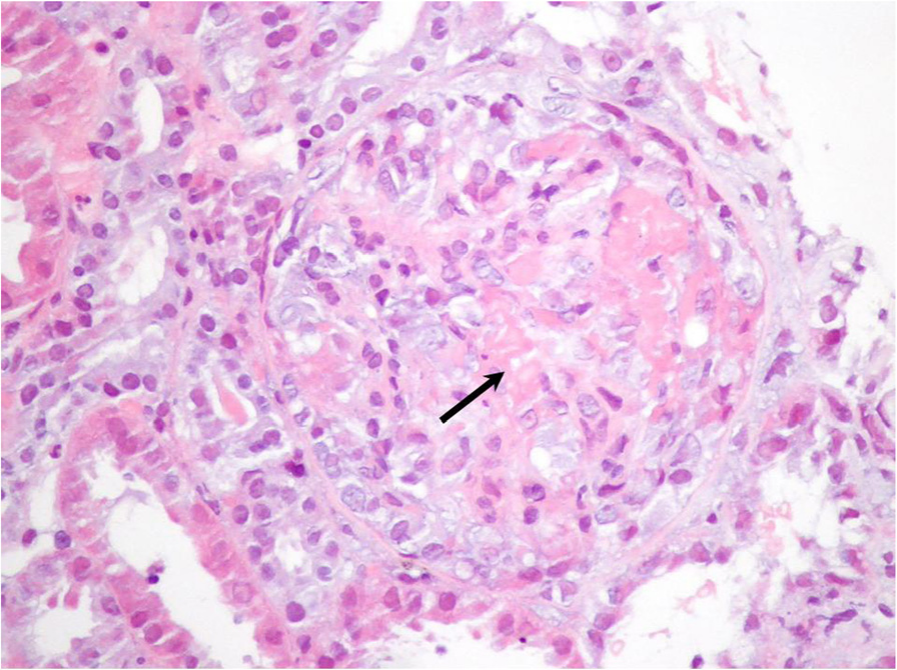
Light microscopy on renal biopsy (hematoxylin eosin stain, ×400). Fibrinoid necrosis (*black arrow*) was seen in glomeruli (19 glomeruli were examined), and there were cellular crescents in 26 % of glomeruli

After 5 days, he was transferred out of our ICU with a good clinical and analytical evolution (Table 1). At the end of the tenth day his general state deteriorated with fatigue and hematuria (327 erythrocytes/L) and in 3 days he developed deterioration of renal function with a serum creatinine level of 4.55 mg/dL and a serum urea level of 189 mg/dL, with recurrence of hemoptysis and anemia, showing a hemoglobin value of 6.1 g/dL (Table 1). His pO_2_ value dropped to 55.5 mmHg with an oxygen saturation of 89.4 % on 50 % oxygen. Immunosuppression with daily prednisolone 1g administered intravenously was initiated. An urgent bronchoscopy was repeated after new transfusion of three blood units, showing no evidence of lesion again. His hemoglobin level rose to 9.1 g/dL after the transfusion. A renal biopsy was performed 24 hours after the bronchoscopy and showed fibrinoid necrosis in glomeruli (19 glomeruli were assessed) and cellular crescents in 26 % of glomeruli (Fig. 4). Immunofluorescence revealed a linear deposition of IgG, compatible with GS. Immunosuppressive therapy with daily cyclophosphamide 120 mg orally was added. Because of renal failure with a serum creatinine level of 5.13 mg/dL, he was transferred to a referral center where he underwent 21 sessions of plasmapheresis and four sessions of hemodialysis. Four weeks after initial presentation, his serum creatinine level was 1.8 mg/dL at the time of his discharge from the reference center. Over the course of 6 months, he completed treatment with daily prednisolone 60 mg orally and daily cyclophosphamide 100 mg orally, maintaining regular follow-up with out-patient nephrology, with currently no need of hemodialysis. No further episodes of hematuria or hemoptysis have been reported so far.

## Discussion

GS typically occurs between the ages of 20 and 30 years and between the ages of 60 and 70 years; the prevalence of the disease is higher in men in the younger age group, as in our case, whereas males and females are equally affected in the older age subgroup [3]. Although the exact cause of GS is unknown, there are certain behaviors and environmental factors that may put people at higher risk. Some factors such as viral respiratory infections, exposure to hydrocarbon fumes, metallic dust, tobacco smoke or substances such as cocaine might be involved [6] and were found in our patient assessment. Symptoms may either start slowly, gradually affecting the lungs and the kidneys, or they may progress rapidly, becoming severe in a matter of days [7]. Constitutional symptoms may precede or be concurrent with pulmonary or renal manifestations. In general, there are substantial variations in the clinical manifestations of patients with GS, with 60 to 80 % of the patients having clinically apparent manifestations of pulmonary and renal disease, 20 to 40 % having renal manifestations alone, and less than 10 % having only pulmonary manifestations [6, 7]. Our case had a bimodal presentation with an initial pulmonary involvement phase that evolved to an almost asymptomatic state followed by a fast pulmonary and renal degradation phase in a matter of 3 days requiring a fast clinical approach.

Diagnosis of GS is made by detection of circulating anti-GBM antibodies on solid phase, and a kidney biopsy provides a definitive diagnosis with pathognomonic findings on direct immunofluorescence of linear deposition of immunoglobulin [8] that was evident in our case; serologic assays for anti-GBM antibodies are valuable for confirming the diagnosis and monitoring the adequacy of therapy [3, 7]. Radioimmunoassays or enzyme-linked immunosorbent assays (ELISAs) for anti-GBM antibodies are highly sensitive (>95 %) and specific (>97 %); immunoassay-based anti-GBM antibody kits showed comparably good sensitivity [9]. In our case, blood samples were processed shortly after collection in our laboratory, which used a EliA™ System (Phadia, Freiburg, Germany). The assay was performed twice on an ImmunoCAP 250 instrument with a sensitivity of 96.4 % and a specificity of 100 %.

At some time during the course of illness, one-third of patients with GS have circulating anti-neutrophil cytoplasmic antibodies (ANCAs) in addition to anti-GBM antibodies. In most cases, ANCAs are detectable several months to years before the development of the disease [3, 6, 7], which was not verified in our patient. In patients with evidence of diffuse alveolar hemorrhage and renal involvement, a kidney biopsy should be considered to identify the underlying cause. Percutaneous kidney biopsy, which is subsequently analyzed by light microscopy, immunofluorescence, and electron microscopy, is the preferred diagnostic procedure [8] and was the cornerstone of our case, performed on time, confirming the diagnosis.

The three principles of therapy include: a) rapid removal of circulating antibodies, primarily by plasmapheresis; b) pharmacological immunosuppression to prevent further production of antibodies; and c) removal of the offending agents that may have triggered the antibody production. Immunosuppressive therapy is required to inhibit antibody production and rebound hypersynthesis, which may occur following discontinuation of plasma exchange [2, 7, 10]. High-dose corticosteroids and cyclophosphamide represent the standard therapy. Initial therapy includes cyclophosphamide 2 mg/kg orally, adjusted to maintain a white blood cell count of approximately 5000 cells, and corticosteroids, which were the choices in this clinical case.

Aggressive therapy with plasmapheresis, corticosteroids, and immunosuppressive agents has dramatically improved the prognosis of GS [11]. Currently, the 5-year survival rate exceeds 80 % and fewer than 30 % of patients require long-term dialysis, as was the case with our patient. Serum creatinine levels greater than 4 mg/dl, oliguria, and the presence of crescents in more than 50 % of glomeruli on renal biopsy predict a worse outcome, with recovery being rare. Until today, no studies on the best treatment of the syndrome have been performed because of the rarity and the sometimes late diagnosis of the syndrome [7]. A correct diagnosis is the first important step to establish correct treatment. In a published case series and in a randomized trial, plasmapheresis was shown to be beneficial in the treatment of GS by the removal of anti-GBM antibodies [12]. Renal transplantation has been used in end-stage renal disease secondary to GS [13]; this was discussed with our patient, but fortunately he had recovery of renal function obviating the need for this option. Although rare, the absence of circulating anti-GBM has already been described [14]. Of interest, our case is similar to the first case reported by Salama *et al*. [14]. The two cases have sex, age group, exposure to risk factors (solvents), and the time of development in common. However, presentation was different; although our case emphasizes the 1-month history of hemoptysis with aggravation the day before admission, Salama *et al*. refer solely to one episode of mild hemoptysis [14]. Furthermore, our patient improved after admission before final clinical deterioration started. In both cases, a renal biopsy was diagnostic. Treatment was also comparable because both patients underwent plasmapheresis and were administered prednisolone orally and cyclophosphamide orally. Evolution was sustainably favorable in the two cases: 8 weeks after initial presentation their patient’s serum creatinine level was 2.5 mg/dL [14] as opposed to our patient who showed a serum creatinine level of 1.8 mg/dL 1 month after initial presentation. In our laboratory, Western blot and biosensor analysis were not available and, thus, further comparisons were not possible.

## Conclusions

GS is a rare syndrome with many types of presentation but it has to be considered in patients presenting with pulmonary hemorrhage or anemia despite the absence of anti-GBM antibodies. A kidney biopsy becomes essential when confronted with this type of presentation, changes in renal function, and clinical suspicion. Although no studies have been performed for treatment approach, with early diagnosis and therapy, survival rates have increased.

The absence of anti-GBM antibodies adds complexity to the diagnostic approach creating an unusual setting in an already rare and complex disease.

## Abbreviations

ANCA, anti-neutrophil cytoplasmic antibody; anti-GBM, anti-glomerular basement membrane; BP, blood pressure; CO_2_, carbon dioxide; CT, computed tomography; CXR, chest X-ray; ELISA, enzyme-linked immunosorbent assay; GBM, glomerular basement membrane; GS, Goodpasture’s syndrome; ICU, intensive care unit; IgG, immunoglobulin G; pO_2_, partial pressure of oxygen.
